# Investigating the Potential Shared Molecular Mechanisms between COVID-19 and Alzheimer’s Disease via Transcriptomic Analysis

**DOI:** 10.3390/v16010100

**Published:** 2024-01-09

**Authors:** Yixian Fan, Xiaozhao Liu, Fei Guan, Xiaoyi Hang, Ximiao He, Jing Jin

**Affiliations:** 1 Department of Physiology, School of Basic Medicine, Tongji Medical College, Huazhong University of Science and Technology, Wuhan 430030, China; 2 Center for Genomics and Proteomics Research, School of Basic Medicine, Tongji Medical College, Huazhong University of Science and Technology, Wuhan 430030, China; 3 Key Laboratory of Vascular Aging of the Ministry of Education, Tongji Medical College, Huazhong University of Science and Technology, Wuhan 430030, China; 4 Hubei Key Laboratory of Drug Target Research and Pharmacodynamic Evaluation, Huazhong University of Science and Technology, Wuhan 430030, China

**Keywords:** COVID-19, Alzheimer’s disease, RNA-seq, gene regulation

## Abstract

SARS-CoV-2 caused the COVID-19 pandemic. COVID-19 may elevate the risk of cognitive impairment and even cause dementia in infected individuals; it may accelerate cognitive decline in elderly patients with dementia, possibly in Alzheimer’s disease (AD) patients. However, the mechanisms underlying the interplay between AD and COVID-19 are still unclear. To investigate the underlying mechanisms and associations between AD progression and SARS-CoV-2 infection, we conducted a series of bioinformatics research into SARS-CoV-2-infected cells, COVID-19 patients, AD patients, and SARS-CoV-2-infected AD patients. We identified the common differentially expressed genes (DEGs) in COVID-19 patients, AD patients, and SARS-CoV-2-infected cells, and these DEGs are enriched in certain pathways, such as immune responses and cytokine storms. We constructed the gene interaction network with the signaling transduction module in the center and identified *IRF7*, *STAT1*, *STAT2*, and *OAS1* as the hub genes. We also checked the correlations between several key transcription factors and the SARS-CoV-2 and COVID-19 pathway-related genes. We observed that *ACE2* expression is positively correlated with *IRF7* expression in AD and coronavirus infections, and interestingly, *IRF7* is significantly upregulated in response to different RNA virus infections. Further snRNA-seq analysis indicates that NRGN neurons or endothelial cells may be responsible for the increase in *ACE2* and *IRF7* expression after SARS-CoV-2 infection. The positive correlation between *ACE2* and *IRF7* expressions is confirmed in the hippocampal formation (HF) of SARS-CoV-2-infected AD patients. Our findings could contribute to the investigation of the molecular mechanisms underlying the interplay between AD and COVID-19 and to the development of effective therapeutic strategies for AD patients with COVID-19.

## 1. Introduction

The global COVID-19 pandemic, caused by SARS-CoV-2 [1,2], has claimed millions of lives and imposed a heavy economic burden on individuals and countries worldwide. The SARS-CoV-2 virus primarily attacks the respiratory system, and COVID-19 patients commonly develop symptoms such as fever, cough, fatigue, and difficulty breathing [3]. However, there is increasing evidence suggesting that COVID-19 patients may also suffer from certain neurological symptoms, including headaches, loss of smell and taste, stroke, and delirium [4,5]. Even after recovering from COVID-19, individuals have reported experiencing cognitive and psychological symptoms, often referred to as “COVID-19 brain fog”, which includes fatigue, poor concentration, and difficulties with memory and sleep [6]. These neurological symptoms have attracted researchers to the topic of evaluating not only the short-term but also the long-term impact of COVID-19 on global public health, including different levels of impairments in the nervous system [7,8,9,10,11].

Older people, especially those with underlying medical comorbidities, such as heart disease, diabetes, cancer, and dementia, are much more susceptible to the severe illness of COVID-19 [12,13,14]. Alzheimer’s disease, the most common form of dementia, causes morbidity and mortality in aging populations worldwide [15]. Some clinical studies have begun to investigate the interactions between AD and COVID-19. Clearly, AD patients are more susceptible to SARS-CoV-2 [14] and also have a higher mortality rate [16,17]. Therefore, some researchers have paid close attention to the impact of COVID-19 on AD [18,19]. Several studies with large cohorts have shown that COVID-19 may elevate the risk level of cognitive impairment and dementia in hospitalized [20,21] patients, and sequelae, such as mental health disorders and neurocognitive disorders in non-hospitalized [22] COVID-19 patients, and it is possible that SARS-CoV-2 infection may accelerate cognitive decline in AD patients [8,23,24].

However, the mechanisms underlying the interplay between AD and COVID-19 remain unclear, and investigating the molecular mechanisms is critical for developing effective therapeutic strategies [25]. One possibility is the direct access of the SARS-CoV-2 virus into the brain, potentially via the olfactory tract [7,26] or through the infiltration of infected immune cells [7,27]. Several genes have been identified as key factors for SARS-CoV-2 infection, including *ACE2* [1], *NRP1* [28], and *BSG* [29] as the viral spike (S) protein docking receptors, and *TMPRSS2* [30], *cathepsin B* (*CTSB*) and *FURIN* [7,31] as the S protein priming protease to facilitate viral cell entry and replication. The olfactory tract has been shown to have a high level of *ACE2* [23,32], and the expression levels of *ACE2* are also elevated in AD patients [33]. Supportive evidence for the infiltration of infected immune cells includes the observation of viral RNA in the macrophages of COVID-19 patients’ bronchoalveolar lavage by single-cell RNA-seq data [27]. Notably, there are no direct autopsy data or cerebrospinal fluid (CSF) studies [5,34] showing the presence of SARS-CoV-2 in the brain so far.

Another possibility concerning the impact of COVID-19 on AD is systemic inflammation and immune dysregulation, either in the central nervous system (CNS) via the overstimulation of the cytokine storm [35,36] or microvascular injury due to perfusion defects [34]. The evidence for this possibility is as follows: (i) the cytokine storm has been observed in severe patients with COVID-19 [35]; (ii) circulating levels of IL-6, IL1b, and TNF were elevated in most COVID-19 patients [7,37]; (iii) Toll-like receptors (TLR), such as TLR2, mediated the inflammation induced by the SARS-CoV-2 spike protein through the NF-kB pathway [38]; (iv) the SARS-CoV-2 spike protein could alter the human blood–brain barrier (BBB) in vitro [39], and the S1 protein could cross the BBB in mice [40]; and (v) the infection of SARS-CoV-2 has increased the circulating concentrations of some nuclear proteins like high mobility group box 1 (HMGB1), while HMGB1 could enter the brain by breaking down the integrity of the BBB, and then induce the innate immune response [41].

**Figure 1:**
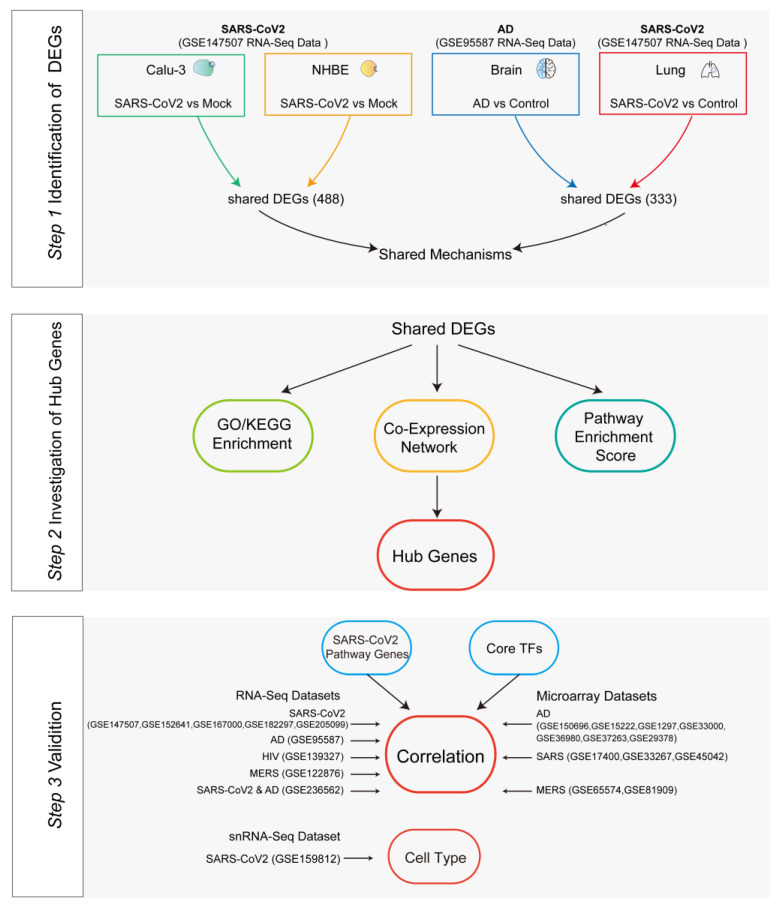
The workflow for identifying critical factors in the interplay between COVID-19 and Alzheimer’s disease (AD). First, the SARS-CoV-2 dataset GSE147507 and the AD dataset GSE95587 were used to find the common differentially expressed genes (DEGs between SARS-CoV-2 cells, COVID-19 patients and AD patients. DEGs were defined as |log2 fold change| ≥ 0.26 and *p*-value < 0.05. Then, classical enrichment analyses, including the GO and KEGG databases, were performed to assess the functional characteristics of the DEGs. Additionally, AD-related pathway enrichment scores in SARS-CoV-2 cells and patients were calculated. Furthermore, the hub genes were obtained using GeneMANIA analysis. Next, the Pearson correlation of hub genes, SARS-CoV-2 pathway-related genes, and SARS-CoV-2-related core transcription factors was performed in multiple datasets. Finally, snRNA-Seq data (GSE159812) were included to determine which cell types exhibited significant enrichment for hub genes, SARS-CoV-2 pathway-related genes, and CoV2-related core transcription factors (TFs).

To explore the molecular mechanisms of SARS-CoV-2 infection, intensive multi-omics datasets, including bulk RNA-seq, single-cell RNA-seq (scRNA-seq), and single-nucleus RNA-seq (snRNA-seq) data, have been generated for COVID-19 patients and SARS-CoV-2-infected cells [9,27,42]. By integrating the large-scale gene expression data of AD [43], this research aims to investigate the underlying mechanisms and interactions between AD progression and SARS-CoV-2 infection (Table 1 and Table S1). As the workflow in Figure 1 shows, we performed a series of bioinformatics analyses in SARS-CoV-2-infected cells, COVID-19 patients, and AD patients. We identified the common differentially expressed genes (DEGs) after SARS-CoV-2 infection in AD patients, and these DEGs are enriched in certain pathways like immune responses, cytokine storms, the Toll-like receptor signaling pathway, and NOD-like receptor signaling pathway. We further constructed the gene interaction network of seven major pathway modules with the signaling transduction module in the center, and *IRF7*, *STAT1*, *STAT2*, and *OAS1* were identified as the hub genes. Interestingly, *IRF7* is a shared DEG in AD patients and also functions as a transcription factor that may regulate the expression of *ACE2*. Importantly, we observed a significant up-regulation of *IRF7* upon various RNA virus infections, and the expression of *ACE2* was positively correlated with *IRF7* expression in both AD and coronavirus infections. Our further analysis using snRNA-seq suggests that NRGN neurons or endothelial cells may be responsible for the increase in *ACE2* and *IRF7* expression after SARS-CoV-2 infection. We observed a positive correlation between *ACE2* and *IRF7* expressions in the hippocampal formation (HF) of SARS-CoV-2-infected AD patients. Our comprehensive analysis may provide supportive evidence of potential brain invasion, as well as strong evidence of neuroinflammation involving immune-related pathways after SARS-CoV-2 infection in AD patients. These findings contribute to the investigation of the molecular mechanisms underlying the interaction between AD and COVID-19 and the development of therapeutic strategies for AD patients with COVID-19.

## 2. Materials and Methods

### 2.1. Data Sources

Gene expression data, including RNA-seq and microarray platforms, were downloaded from public databases, as summarized in Table 1. Briefly, the major datasets included GSE147507 [42] with 2 CoV2 cell lines (Calu3, NHBE) and COVID-19 lungs (COVID-19Lung); GSE95587 [43] was used for 33 fusiform gyrus controls and 84 AD cases. Additionally, we collected AD datasets GSE150696 [44], GSE15222 [45], GSE1297 [46], GSE33000 [47], GSE36980 [48], GSE37263 [49], and GSE29378 [50], along with COVID-19 datasets GSE152641 [51], GSE167000 [52], GSE182297 [53], GSE188847 [54], and GSE205099 [55]. For SARS, the datasets GSE17400 [56], GSE33267 [57], and GSE45042 [58] were used. MERS datasets included GSE122876 [59], GSE65574 [60], and GSE81909 [61]. The HIV dataset GSE139327 [62] and the AD dataset with SARS infection GSE236562 [63] were also incorporated. Additionally, snRNA-Seq data for 30 samples from GSE159812 [9] were downloaded from GEO. Detailed information about these datasets is provided in Table S1. The Alzgset (AD-related genes set) and 68 enriched pathways for Alzgset were collected according to the published literature [64].

### 2.2. Processing of Gene Expression Data

For RNA-seq data, if the raw fastq files were available, the raw reads were mapped to the human reference genome (hg38) using the STAR aligner v2.5.1b [65] with parameters, as suggested by the ENCODE guidelines [66] and the quantification of gene counts for each gene was obtained via the --quantMode parameter of STAR. The read count matrix of AD was obtained from GSE147507. Microarray data were normalized and log2-transformed using the limma R package (version 3.44.1). Differential expression analysis was conducted using the DESeq2 R package (version 1.28.1) [67] with a cutoff *p*-value < 0.01 and |log2(fold-change)| > 0.26 to identifyDEGs. All significant DEGs were visualized in a volcano plot generated using the R package ggplot2 (3.4.4) [68]. Venn diagrams were created using the vennDiagram package (v1.7.3) in R [69]. The gene counts were normalized using the DESeq2 estimateSizeFactorsForMatrix method. The normalized count data from DESeq2 was processed as log2(DESeq2NorCounts + 1) and then standardized using the min–max normalization method for visualization in a heatmap using the ComplexHeatmap package (v2.12.0) [70] in R.

### 2.3. snRNA-Seq Data Analysis

The gene expression matrix of snRNA-seq data was imputed in the Seurat R package (version 4.0.0) [71]. PCA was used to cluster the cells with the top 10 principal components by running the FindNeighbors function with reduction = “pca” and dims = 1:10. The FindClusters function was used to identify clusters with a resolution = 0.3. The differential analysis of cell subgroups was performed using the Findmarker function provided by Seurat. Cell types were annotated for each cell type from a user-provided mtx file.

### 2.4. Gene Enrichment Analysis with Functional Annotation

To identify enriched GO terms for DEGs, we performed the pathway and process enrichment analyses for the selected gene list using Metascape (metascape.org, accessed on 13 November 2023) [72] with a *p*-value of less than 0.01 using the GO biological process module. Gene pathway analysis was further carried out using GeneAnalytics (geneanalytics.genecards.org, accessed on 21 November 2021) [73] via the GeneCards Suite.

### 2.5. Calculation of Pathway Enrichment Score (PES)

To determine the enrichment of AD-related pathways in CoV2 samples, we calculated a pathway enrichment score (PES) for each AD-related pathway. For each pathway gene set in 68 enriched pathways collected as previously described [64], we calculated the overlap of the pathway gene set with each CoV2 group: *N = (AD pathway Genes ∩ each CoV2 DEGs)*. An example of AD-related PES in Calu3 was calculated using the following formula:

$$\mathrm{PES}=\frac{N(AD\;pathway\;Gene\;Number\;\cap \;Calu3\;DEGs)}{N\left(AD\;pathway\;Gene\;Number\right)\times (N(Calu3\;DEGs)/N(Calu3\;DEGs\;\cap \;AD\;DEGs))}$$

The significance of PES was tested using the chi-squared test. PESs in lung COVID-19, NHBE, and Calu3 were calculated separately, filtering via the PES > 2 and *p*-value < 0.05.

### 2.6. Interaction Network of Up-Expressed DEGs upon SARS-CoV-2 Infection

The gene–gene interaction network was analyzed and visualized using the GeneMANIA v3.5.2 [74] plugin of Cytoscape v3.8.0 [75]. The co-expression database was constructed with the expression of COV2 common up-expressed DEGs. The publicly available database of GeneMANIA was used for co-localization, genetic interactions, pathways, physical interactions, and predicted and shared protein domains. The gene interaction network was also verified using STRING v11.0 (https://string-db.org/, accessed on 13 March 2021) [76].

### 2.7. Transcription Factor Binding Sites (TFBS) Analysis with MEME

TFBS from the TRANSFAC database (v) (http://genexplain.com/transfac/, accessed on 7 June 2020) [77] was used to construct position weight matrices for the motif prediction. The promoter for each gene was defined as 1.5 kb upstream of TSS and 500 bp downstream of TSS. Local motif identification was performed via MAST (Motif Alignment and Search Tool) programs (http://www.sdsc.edu/MEME/meme/website/html, accessed on 13 January 2023) from the MEME software suite with default parameters [78].

### 2.8. Statistical Analysis

All statistical analyses were performed with the R statistical software using the Wilcoxon rank–sum test or the Chi-squared test.

## 3. Results

### 3.1. Identification of DEGs Shared between AD and COVID-19 Patients

**Figure 2:**
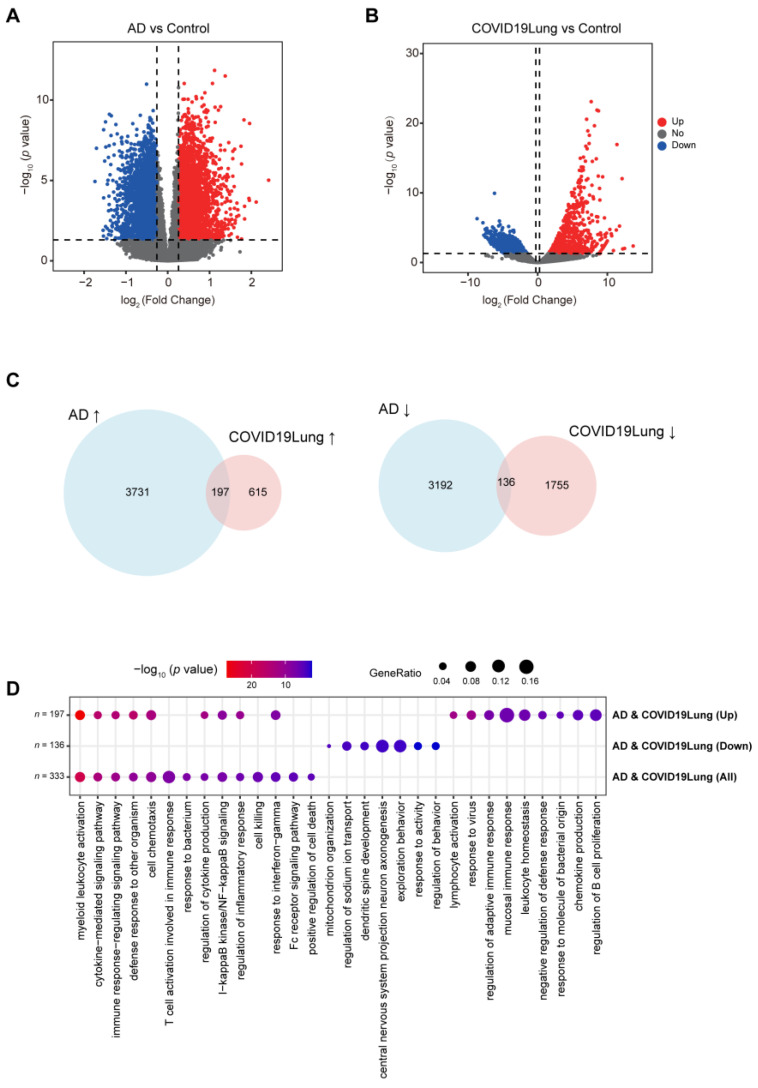
DEGs are shared in AD and COVID-19 patients. (**A**) The volcano plot shows the DEGs in AD patients. (**B**) The volcano plot shows the DEGs in the lung tissues of COVID-19 patients. Red dots are genes that are up-expressed, blue dots are genes that are down-expressed, and gray dots are genes that are not significantly differentially expressed. (**C**) The Venn diagram shows the shared DEGs that are up-expressed (up) and down-expressed (down) in both AD patients and COVID-19 patients. (**D**) GO analysis identified enriched gene clusters for up-expressed DEGs (*n* = 197) and down-expressed DEGs (*n* = 136) and combined all shared up-/down-regulated DEGs (*n* = 333) in both AD and COVID-19 patients.

In our investigation into the potential increased susceptibility of AD patients to COVID-19 infection, we analyzed differential gene expression in both conditions. In the brain tissue of AD patients [43], compared to normal brains, we identified 3928 genes that were upregulated and 3328 genes that were downregulated (Figure 2A). Similarly, in the lung tissue of COVID-19 patients [42], we found 812 genes that were upregulated and 1891 genes that were downregulated (Figure 2B). Notably, there was a significant overlap in gene expression changes between the two conditions: 197 genes were commonly upregulated in both AD and COVID-19 patients, while 136 genes were commonly downregulated (Figure 2C). These shared gene expression patterns could indicate a biological link between AD and increased vulnerability to COVID-19 infection.

Subsequently, to identify the intersecting genes in AD and COVID-19 patients, we conducted a comprehensive GO BP and KEGG pathway enrichment analysis (Figure 2D and Figure S1A). For the genes that were upregulated in both AD and COVID-19, the enrichment analysis revealed a significant association with pathways related to immune response and inflammation. Specifically, these genes were predominantly enriched in pathways such as myeloid leukocyte activation, cytokine-mediated signaling, the immune response-regulating signaling pathway, the defense response to other organisms, and cell chemotaxis. This suggests a possible shared mechanism involving heightened immune and inflammatory responses in both AD and COVID-19. Conversely, the downregulated intersecting genes were mainly enriched in pathways related to mitochondrial function, ion transport, neural development, and behavior (Figure 2D). The manipulation of mitochondria via SARS-CoV-2 could induce mitochondria dysfunction and then increase mitochondria-derived double-membrane vehicles in which the virus can hide and replicate [79]. Mitochondria have been reported to be involved in the process of inflammation in both innate and adaptive immunity [80]. When we combined the shared upregulated and downregulatedDEGs, the most enriched GO term emerged as myeloid leukocyte activation, alongside significant enrichment in the homeostasis of the number of cells pathway. Moreover, cell death is well known to play an important role in AD progression [64]. This indicates the potential common pathway of neural and mitochondrial dysfunction in both conditions. These findings are consistent with our proposal that the altered enriched pathways involved in immune responses, cell death, and mitochondrial dysfunction upon SARS-CoV-2 infection are altered in similar patterns in AD, which may accelerate cognitive decline in AD patients.

### 3.2. Identification of DEGs Shared between SARS-CoV-2-Infected Calu-3 Cells and NHBE Cells

**Figure 3:**
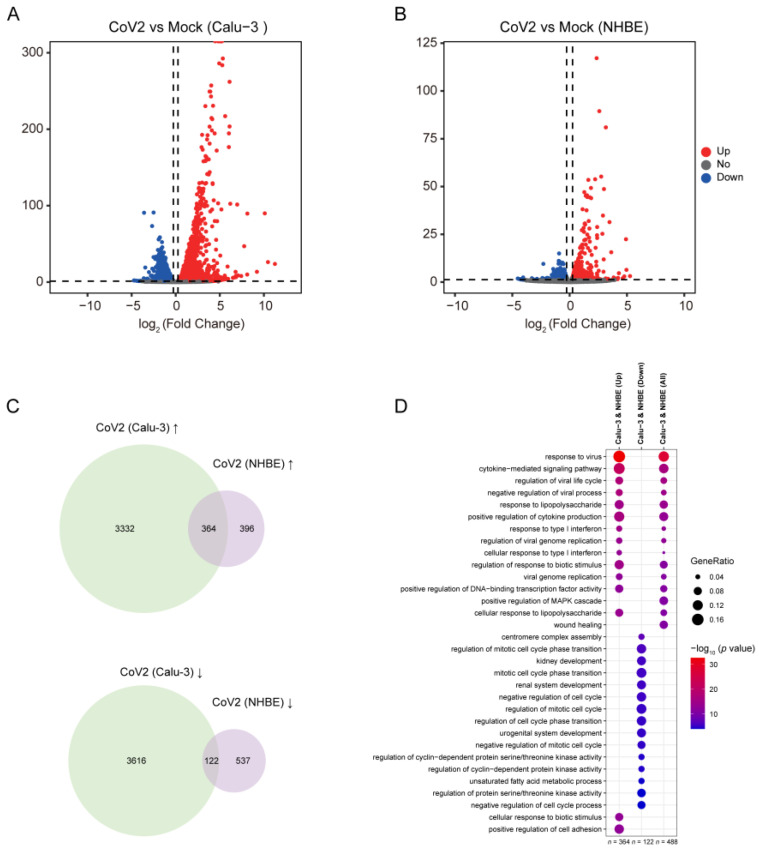
DEGs are shared in AD and COVID-19 patients. (**A**) The volcano plot shows the DEGs in SARS-CoV-2-infected Calu-3 cells. (**B**) The volcano plot shows the DEGs in SARS-CoV-2-infected NHBE cells. Red dots are genes that are up-expressed, blue dots are genes that are down-expressed, and gray dots are genes that are not significantly differentially expressed. (**C**) The Venn diagram shows the shared DEGs that are up-expressed (up) and down-expressed (down) in both SARS-CoV-2-infected Calu-3 cells and NHBE cells. (**D**) GO analysis identified enriched gene clusters for up-expressed DEGs (*n* = 364) and down-expressed DEGs (*n* = 122) and combined all shared up-/down-regulated DEGs (*n* = 488) in both SARS-CoV-2-infected Calu-3 cells and NHBE cells.

In our comprehensive study to understand the biological pathways implicated in the SARS-CoV-2 infection, we further conducted the analysis of gene expression changes in Calu-3 cells, human epithelial cells located on the pulmonary surface, and normal human bronchial epithelial (NHBE) cells following SARS-CoV-2 infection. Our findings revealed significant alterations in the gene expression in response to the virus in both cell types. In the Calu-3 cell line, a substantial number of genes were affected, with 3696 genes showing upregulation and 3738 genes that were downregulated (Figure 3A). In NHBE cells, the impact was also notable, albeit on a smaller scale, with 760 genes upregulated and 659 genes downregulated after infection (Figure 3B). We discovered that there were 364 genes that were consistently upregulated and 122 genes that were consistently downregulated in both Calu-3 and NHBE cells (Figure 3C). The commonly upregulated genes were primarily enriched in pathways that play pivotal roles in viral response mechanisms (Figure 3D and Figure S1B). These include the response to the virus, the cytokine-mediated signaling pathway, the regulation of viral processes, and the response to type I interferon. Conserved signaling pathways observed across different cell lines infected with SARS-CoV-2, specifically cytokine-mediated signaling pathways and immune responses, may trigger systemic inflammation and a cytokine storm, resulting in a significant increase in pro-inflammatory cytokines. There is evidence suggesting that neuroinflammation can impact cognitive functions and accelerate the progression of neurodegenerative diseases [81]. This implies that the inflammatory response induced by SARS-CoV-2 could potentially exacerbate the progression of AD. Conversely, the downregulated genes in both cell types were significantly enriched in pathways associated with cell cycle control and metabolic processes (Figure 3D). Key among these were the regulation of the mitotic cell cycle phase transition, the regulation of the mitotic cell cycle, the regulation of cyclin-dependent protein serine/threonine kinase activity, and the unsaturated fatty acid metabolic process. SARS-CoV-2’s effect on cellular functions, such as the cell cycle and metabolic processes, as observed in SARS-CoV-2-infected lung cells, might also occur in neural cells [82,83]. Since AD already involves disruptions in neural cell functions, additional stress from SARS-CoV-2 could exacerbate these disruptions.

### 3.3. Changes in the Expression of Top AD DEGs in SARS-CoV-2-Infected Cells and in COVID-19 Patients

**Figure 4:**
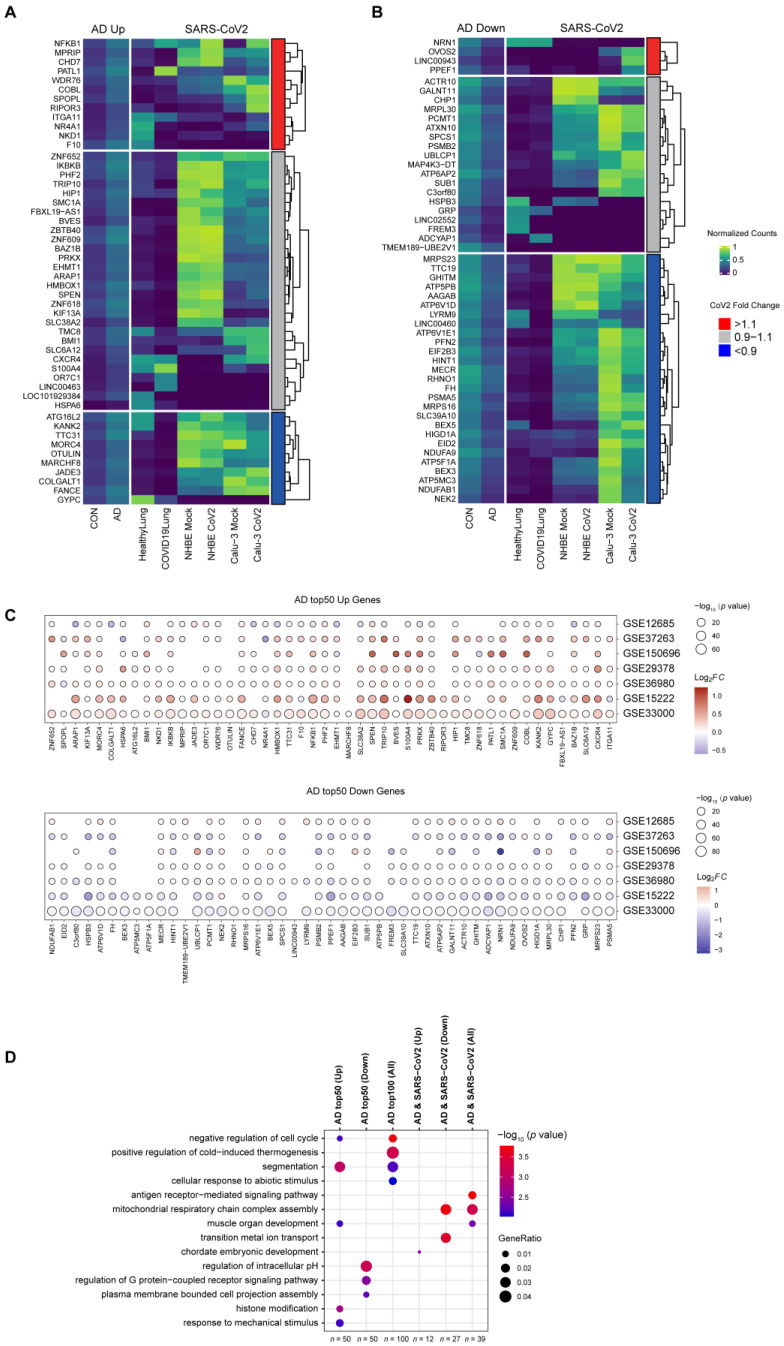
Changes in the expression of top AD DEGs in cells infected with SARS-CoV-2 and in COVID-19 patients (**A**,**B**) Heatmaps show the min–max normalized gene expression from DESeq2 changes in COVID-19 patients and SARS-CoV-2-infected Calu-3 and NHBE cells for the top 50 up-expressed (**A**) and down-expressed (**B**) DEGs in AD patients. The red bar indicates the up-expressed genes (fold change > 1.1) upon SARS-CoV-2 infection in at least 2 of 3 groups (COVID-19 patients, Calu-3, or NHBE); the blue bar indicates the down-expressed genes (fold change < 0.9) upon SARS-CoV-2 infection in at least 2 of 3 groups. (**C**) Differential expression patterns of the top 50 upregulated and downregulated genes from dataset GSE95587 across multiple datasets. The size of each point correlates with the *p*-value. The color of the points denotes the fold change in differential expression. (**D**) GO analysis identified enriched gene clusters for TOP 50 up- and down-expressed DEGs in AD patients, the shared up-expressed (*n* = 12, red bar in (**A**)), down-expressed (*n* = 27, blue bar in (**B**)), and up-/down-expressed (*n* = 39, red bar in (**A**) + blue bar in (**B**)) genes with a similar expression change pattern in AD patients, COVID-19 patients and SARS-CoV-2-infected Calu-3 and NHBE cells.

To investigate how SARS-CoV-2 infection affects AD progression, we also studied how the expression profiles of the top AD DEGs change after SARS-CoV-2 infection. We first identified the top 50 up- and 50 down-expressed DEGs in AD patients. We then investigated the expression profiles of these top up- and down-regulated AD DEGs in COVID-19 patients, and also in NHBE and Calu-3 cells, before and after SARS-CoV-2 infection (Figure 4A,B). This analysis was extended to include an examination of the top 50 upregulated and top 50 downregulated genes across various datasets. Remarkably, we found that the expression patterns of these top differentially expressed genes in other datasets were similar to the patterns observed in the GSE95587 dataset. This consistency across multiple datasets underscores the potential significance of these genes in AD pathology (Figure 4C). We also performed GO and KEGG analysis on these top up- and down-expressed DEGs. The 50 up-expressed DEGs, are enriched in segmentation, histone modification, the negative regulation of the cell cycle, muscle organ development, and the response to a mechanical stimulus (Figure 4D and Figure S1C), indicating the possible role of cell division in AD progression. The 50 down-expressed DEGs are enriched in the regulation of intracellular pH, the regulation of the G protein-coupled receptor signaling pathway, and plasma membrane-bounded cell projection assembly, highlighting the role of hypoxia in AD pathogenesis. Combining the up- and down-expressed DEGs, we found that the positive regulation of cold-induced thermogenesis and cellular response to abiotic stimulus are also enriched. Taken together, these enriched GO terms are consistent with the current knowledge that inflammatory responses and apoptosis play important roles in AD progression and also support the hypothesis that hypoxia could lead to chronic activation and the recruitment of pro-inflammatory immune cells [84].

Among the top 50 up-regulated AD DEGs, 12 showed a similar up-expression pattern upon SARS-CoV-2 infection (Figure 4A), with more than 10% up-expressed in at least 2 of the 3 conditions (COVID-19 patients or 2 cell lines). *NFKB1* shows almost the same expression pattern in AD patients, COVID-19 patients, and in NHBE and Calu-3 cells, highlighting the important role in both AD and COVID-19 progression. These 12 genes are enriched in GO in terms of chordate embryonic development (Figure 4D). Similarly, among the top 50 down-regulated AD DEGs, 54% of them (27 genes) are down-expressed upon SARS-CoV-2 infection. *NDUFA* (*NDUFA9*, *NDUFAB1*), *ATF* (*ATP5F1A*, *ATP5PB*, *ATP5MC3*, *ATP6V1D*, *ATP6V1E1*), *PSMA* (*PSMA1*, *PSMA5*), and *MRPS* (*MRPS16*, *MRPS23*) families are enriched in these 27 genes. The most enriched GO term for these 27 genes is the mitochondrial respiratory chain complex assembly, which highlights the critical role of mitochondria in AD and COVID-19 progression [79]. When the common 12 up-expressed and 27 down-expressed DEGs are combined, these genes are enriched in a new GO term of antigen receptor-mediated signaling pathway, which again supports the observation that inflammatory responses play an essential role in both AD progression and SARS-CoV-2 infection.

### 3.4. Changes in the Expression of the Most Common DEGs Associated with SARS-CoV-2 Infection in AD Patients

**Figure 5:**
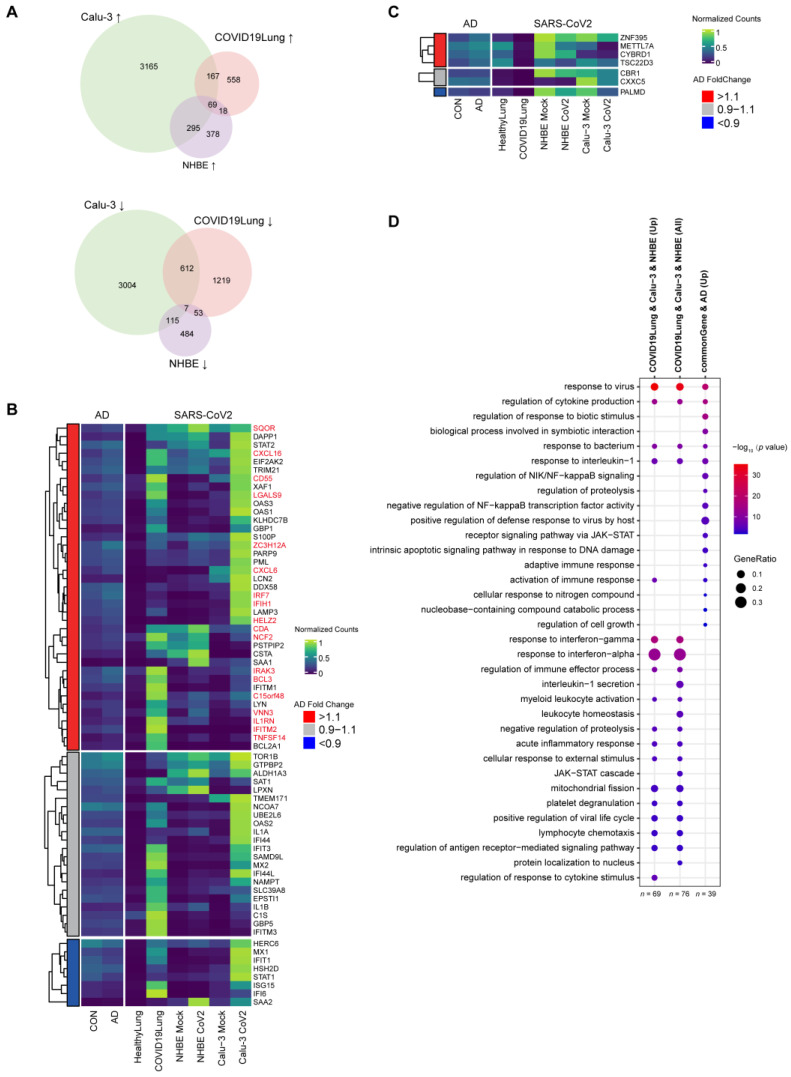
AD expression changes in the most common DEGs in SARS-CoV-2-infected cells and COVID-19 patients. (**A**) Venn diagram shows the shared DEGs that are up-regulated (left) and down-regulated (right) in COVID-19 patients, SARS-CoV-2-infected NHBE, and Calu-3 cells. (**B**,**C**) Heatmaps show the min–max normalized gene expression changes in AD patients for the common 69 up-regulated (**B**) and 7 down-regulated (**C**) DEGs observed in COVID-19 patients and SARS-CoV-2-infected NHBE and Calu-3 cells. Gene symbols highlighted in red represent the 18 DEGs that are commonly up-regulated in AD patients, COVID-19 patients, and SARS-CoV-2-infected Calu-3 and NHBE cells. (**D**) GO analysis identified enriched gene clusters for 3 groups of shared DEGs, including (1) all shared up-regulated DEGs in COVID-19 patients and SARS-CoV-2-infected Calu-3 and NHBE cells (*n* = 69); (2) all shared up-regulated and down-regulated DEGs in COVID-19 patients and SARS-CoV-2-infected Calu-3 and NHBE cells (*n* = 76); (3) and the up-regulated DEGs in COVID-19 patients and SARS-CoV-2-infected Calu-3 and NHBE cells, which are also up-expressed in AD (10% increase, *n* = 39).

We then asked what the expression levels of SARS-CoV-2 DEGs are in AD patients. We first compared the DEGs in SARS-CoV-2-infected Calu-3 and NHBE cells as well as in COVID-19 patients to identify the common DEGs upon SARS-CoV-2 infection (Figure 5A). We found 69 common DEGs that were up-expressed (left) and 7 common DEGs that were down-expressed (right) after SARS-CoV-2 infection. Subsequently, we checked the expression levels of these common DEGs in AD patients (Figure 5B,C). Among the 69 up-expressed genes, 39 genes were also identified to be up-expressed (fold change, FC > 1.1) in AD patients (Figure 5B), and 18 of these genes were also AD DEGs considering more stringent criteria (gene symbols in red, FC > 1.2, and *p*-value < 0.01). We also performed GO analysis on the common DEGs upon SARS-CoV-2 infection and found enrichment in the regulation of cytokine production and the response to interferon-alpha (Figure 5D and Figure S1D), highlighting the important roles of interferons in SARS-CoV-2 infection, as supported by a recent study [85]. Further GO analysis on the 39 genes sharing a similar up-regulated pattern revealed enrichment in the biological process involved in symbiotic interaction, the positive regulation of the defense response to the virus by the host, the receptor signaling pathway via JAK-STAT, adaptive immune response, and regulation of cell growth (Figure 5D). Among the 7 down-regulated genes, *PALMD* was also identified to be down-expressed in AD patients (FC < 0.9, Figure 5C).

### 3.5. IRF7 Plays Key Roles in Pathways Involved in Signaling Transduction in Both AD and SARS-CoV-2 Infection

**Figure 6:**
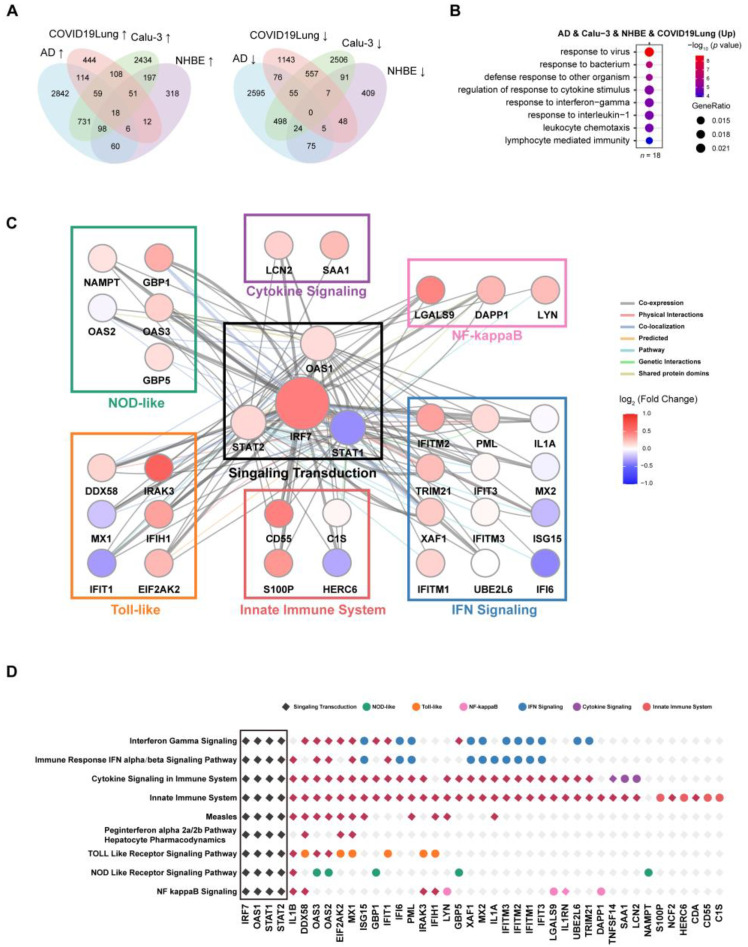
*IRF7* plays a key role in pathways involved in signaling transduction in both AD and SARS-CoV-2 infection. (**A**) Venn diagram shows shared DEGs that are up-regulated (left) and down-regulated (right) in AD, COVID-19 patients, and SARS-CoV-2-infected Calu-3 and NHBE cells. (**B**) GO analysis identified enriched gene clusters for the shared up-regulated DEGs in AD, COVID-19 patients, and SARS-CoV-2-infected Calu-3 and NHBE cells (*n* = 18); (**C**) Network showing the up-expressed genes involved in signaling transduction with annotated functions. The network was constructed based on the common up-expressed genes (*n* = 69) upon SARS-CoV-2 infection in Calu-3 cells, NHBE, or COVID-19 patients. Nodes correspond to genes; the color of a node indicates the log2 fold-change between AD patients versus the controls. Edges are inferred by GeneMANIA and correspond to physical interactions, colocalization, or co-expression. The remaining genes, which are part of this signature but with unknown/unrelated functions, can be found in Figure 5B. (**D**) The enriched pathways and involved genes with the notation of functional groups in the network are shown in (**C**). The black diamonds indicate the four genes that appeared in all pathways, marked as a group of signaling transduction; the colored diamond indicates the gene belonging to the corresponding pathway, and the colored circle indicates the gene belonging to the selected functional groups in (**C**).

We then identified the most common DEGs in AD patients, COVID-19 patients, and SARS-CoV-2-infected Calu-3 and NHBE cells (Figure 6A). In total, 18 genes were identified as common DEGs across AD patients, COVID-19 patients, and CoV2-infected Calu-3 and NHBE cells. These 18 essential genes include *IRF7*, *IFIH1*, *IFITM2*, etc., and GO and KEGG analysis indicate that these DEGs are enriched in the viral process, response to the virus, the regulation of response to cytokine stimulus, and leukocyte homeostasis, which again highlights the important roles of immune responses in both AD progression and SARS-CoV-2 infection (Figure 6B and Figure S1E).

In order to identify the essential hub genes in the interaction network, we classified all 69 up-expressed common DEGs upon SARS-CoV-2 infection into different pathways on the basis of their annotated functions (Figure 6C,D and Figure S2). We found that some genes could be classified into several different pathways, such as *IRF7*, *OAS1*, *STAT1*, and *STAT2*, indicating their multifaceted roles (Figure 6D). According to the gene’s physical interactions, colocalization, and co-expression, the gene interaction network with pathway annotation was constructed (Figure 6C). We found 7 major pathway modules, as shown in Figure 6C, with the signaling transduction module located in the center of the network. *IRF7*, *STAT1*, *STAT2*, and *OAS1* are involved in all 9 enriched pathways (Figure 6D), indicating their important roles in regulating the gene expression network. Based on these observations, *IRF7*, *STAT1*, *STAT2*, and *OAS1* have been identified as the essential genes, and the signaling transduction module is the core of the network. Among the four genes, *IRF7* is the most up-regulated gene, while *STAT1* is down-regulated in AD, suggesting that *IRF7* plays a key role in the interaction network of regulating gene expression in both AD progression and SARS-CoV-2 infection. In the network, *IRF7* may regulate immune responses through the following several nodes: *LCN2* in the cytokine signaling module, *LGAS9* and *DAPP1* in the NF-kappaB signaling module, *IFITM2*, *TRIM21* in the IFN signaling module, *CD55* and *S100P* in the innate immune system module, *IRAK3* and *IFIH1* in the Toll-like receptor signaling module, or *NAMPT* and *OAS3* in the NOD-like receptor signaling module.

### 3.6. IRF7 Is Significantly Up-Regulated upon Different RNA Virus Infections, and the Expression of ACE2 Is Positively Correlated with IRF7 Expression in Both AD and Coronavirus Infections

**Figure 7:**
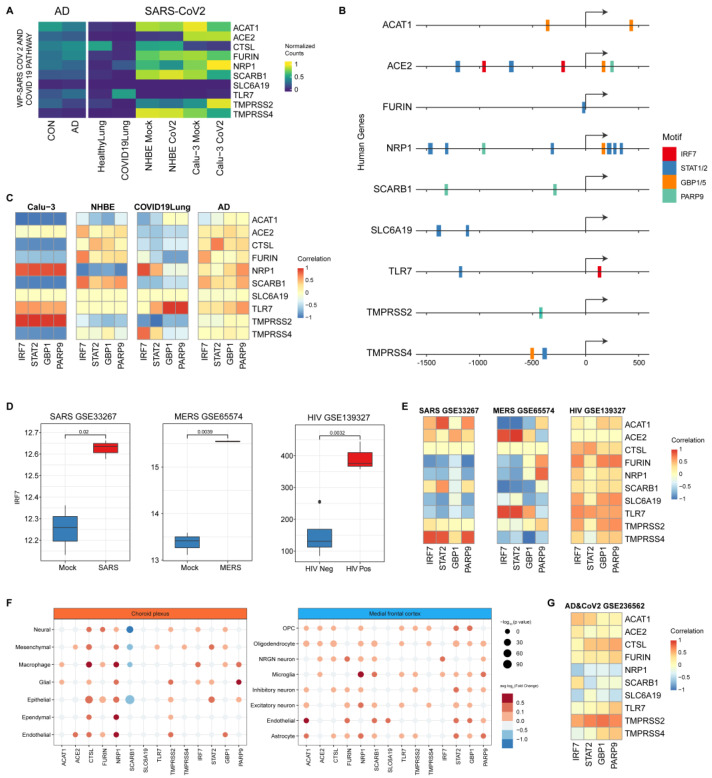
*IRF7* is significantly up-regulated in response to different RNA virus infections, and the expression of *ACE2* is correlated with *IRF7*. (**A**) Heatmaps show the min–max-normalized gene expression from DESeq2 changes in patients AD and COVID-19 lung and SARS-CoV-2-infected cells (Calu-3 and NHBE) for the SARS-CoV-2 and COVID-19 pathway-related genes. (**B**) Selected major TFs binding to the promoter regions (−1500 to 500 bps from TSS) in the SARS-CoV-2 and COVID-19 pathway-related genes predicted by MAST. The arrows indicate the direction of transcription, showing the pathway of RNA polymerase along the DNA strand for RNA synthesis. (**C**) Correlation of gene expression between the SARS-CoV-2 and COVID-19 pathway-related genes and 4 major TFs in patients (AD and COVID-19 lung) and SARS-CoV-2 infected cells (Calu-3 and NHBE). (**D**) *IRF7* is up-regulated upon infections with different RNA viruses, including SARS, MERS, and HIV. (**E**) Correlation of gene expression with the same genes in (**C**) but for SARS, MERS, and HIV. (**F**) Expression changes in the SARS-CoV-2 and COVID-19 pathway-related genes and 4 major TFs in different cell types in human brains upon SARS-CoV-2 infection. (**G**) Correlation of gene expression with the same genes in (**C**) but for SARS-CoV-2-infected AD patients.

We further investigated the genes involved in the SARS-CoV-2 and COVID-19 pathway (WP4846) retrieved from the WikiPathways database [86]. We first analyzed the expression levels of these genes in both AD patients, COVID-19 patients, and SARS-CoV-2-infected Calu-3 and NHBE cells (Figure 7A). We found that *ACE2* and *TLR7* are expressed at low levels in healthy brains, slightly increased in AD patients, while *TMPRSS2/4* and *SLC6A19* are extremely low-expressed, with nearly no expression, in AD patients. The expression levels of *ACAT1* and *CTSL* show opposite trends in AD patients and COVID-19 patients.

To identify transcription factors (TFs) that may bind to the SARS-CoV-2 and COVID-19 pathway-related genes, we analyzed the upstream regulatory regions of these genes to predict transcription factor-binding sites (Figure 7B). We found 6 transcription factors among the 69 up-regulated genes in COVID-19 patients and SARS-CoV-2-infected cells (Figure 5B) that may bind to these genes, including genes in the *STAT* family (*STAT1/2*), *GBP* family (GBP1/5), *IRF7* and *PARP9* This highlights the essential regulatory roles of genes in the *STAT* and *IRF* family in SARS-CoV-2 infection, as evidenced by the hub genes of *STAT2* and *IRF7* in the network shown in Figure 6C. We then investigated the correlation between the expression of the SARS-CoV-2 and COVID-19 pathway-related genes and 4 major TFs that are up-regulated in AD (Figure 7C and Figure S3). The *ACE2* expression was positively correlated with the expression of *IRF7* in Calu-3, NHBE, and AD patients, and the expression of *TMPRSS2* was positively correlated with the expression of *IRF7* in Calu-3 and AD patients (Figure 7C). Considering *IRF7*’s role as both a DEG in AD and SARS-CoV-2 infection and also as a transcription factor that might bind with the human *ACE2* promoter, which is very important for viral penetration into cells [87], we suspected that *IRF7* might be a key factor for SARS-CoV-2 infection and AD progression. To study the function of *IRF7* in AD progression and SARS-CoV-2 infection, we first checked the expression changes in *IRF7* in other independent AD and SARS-CoV-2 datasets (Table 1). As expected, *IRF7* expression is significantly increased in AD patients, SARS-CoV-2 infected cells, and also in patients infected with SARS-CoV-2 (Figure S4).

We then extended the analysis to other RNA viruses, and the results indicated that the expression of *IRF7* is also significantly increased in other RNA virus infections (Figure 7D), including SARS (*p *= 0.02), MERS (*p *= 0.0039) and HIV (*p *= 0.0032). Next, we checked the correlation between expressions of the SARS-CoV-2 and COVID-19 pathway-related genes and 4 major TFs in these datasets (Figure 7E) and observed that *ACE2* expression is positively correlated with *IRF7* expression in both SARS and MERS. The correlation between *ACE2* and *IRF7* expressions was not observed in white cells because *ACE2* is not expressed in HIV infection (Figure S5). It can be noted that the positive correlation of *ACE2* and *IRF7* expression is not observed in some other independent datasets (Figure S5). Furthermore, we investigated the expression changes in the SARS-CoV-2 and COVID-19 pathway-related genes and 4 major TFs in different cell types in the human brain upon SARS-CoV-2 infection. The snRNA-seq analysis indicates that NRGN neurons or endothelial cells may be responsible for the elevated expression of *ACE2* and *IRF7* after SARS-CoV-2 infection (Figure 7F). Lastly, we observed the positive correlation between *ACE2* and *IRF7* expressions both in the frontal cortex of COVID-19 patients (Figure S5) and in the hippocampal formation (HF) of SARS-CoV-2-infected AD patients (Figure 7G). Taken together, these results support our hypothesis that *IRF7* may regulate *ACE2* expression and could be a critical factor for SARS-CoV-2 infection; *IRF7* may modulate the immune responses in AD progression and other RNA virus infections.

### 3.7. Identifications of the Most Enriched Pathways in Both AD and SARS-CoV-2 Infection

**Figure 8:**
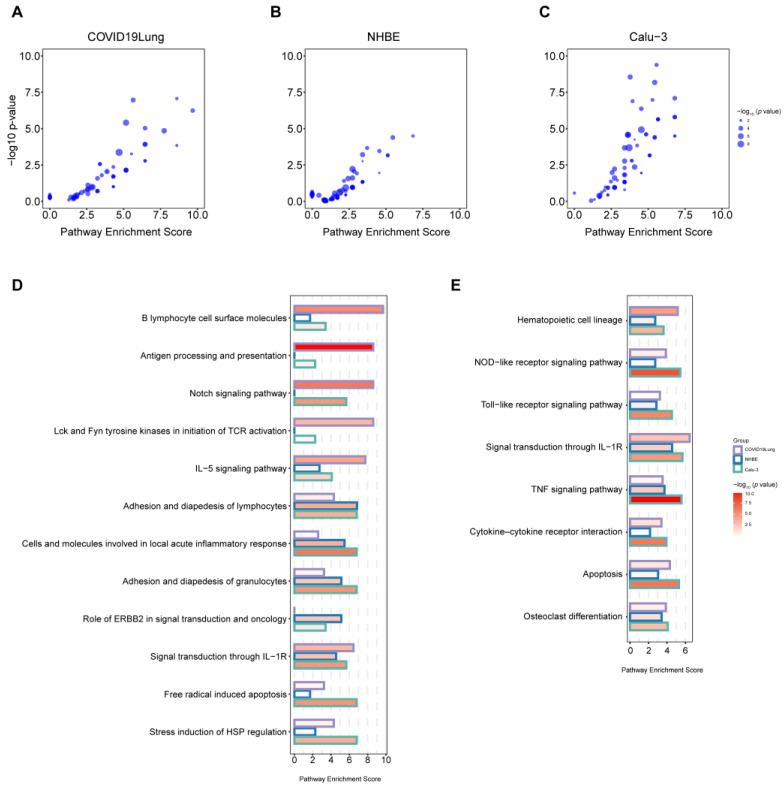
The enriched pathways in both AD and SARS-CoV-2 infection. (**A**–**C**) The scatter plot shows the pathway enrichment scores of DEGs in COVID-19 patients (**A**) and SARS-CoV-2-infected Calu-3 (**B**) and NHBE cells (**C**) for the enriched pathways in AD patients identified by [64]. (**D**) The top enriched pathways upon SARS-CoV-2 infection (each top 5 enriched pathway in COVID-19 patients, or SARS-CoV-2-infected Calu-3 and NHBE cells). (**E**) The most common enriched pathways are shared in both AD and SARS-CoV-2 infection.

An alternative approach to study the impact of SARS-CoV-2 infection on AD patients is to use AD DEGs to obtain the enriched pathways in AD and then check whether the genes that are included in these pathways are DEGs in SARS-CoV-2 infection. To this end, we calculated the pathway enrichment scores (PES) of DEGs in COVID-19 patients (Figure 8A) and SARS-CoV-2-infected NHBE (Figure 8B) and Calu-3 (Figure 8C) cells for the enriched pathways in AD patients in a recent study [64]. For COVID-19 patients, the top 5 most enriched pathways are B lymphocyte cell surface molecules (PES = 9.68, *p *= 5.71 × 10^−7^), Lck and Fyn tyrosine kinases for the initiation of TCR activation, the Notch signaling pathway, antigen processing and presentation (PES = 8.60, *p *= 0.00014), and IL-5 signaling pathway (PES = 7.74, *p *= 1.40 × 10^−5^; Figure 8D, Table S2). In SARS-CoV-2-infected NHBE and Calu-3 cells, the three most enriched pathways are the lymphocytes (Calu-3 PES = 6.80, *p* = 3.21 × 10^−5^; NHBE PES = 6.83, *p *= 3.20 × 10^−5^), cells and molecules involved in local acute inflammatory response (Calu-3 PES = 6.80, *p *= 8.13 × 10^−8^; NHBE PES = 5.46, *p *= 4.07 × 10^−5^), and the adhesion and diapedesis of granulocytes (Calu-3 PES = 6.80, *p *= 1.59 × 10^−6^; NHBE PES = 5.12, *p *= 0.00068; Figure 8D, Table S2). The other two or three of the top five most enriched pathways for NHBE cells are the role of ERBB2 in signal transduction and oncology (PES = 5.12, *p *= 0.00068), Signal transduction through IL-1R, and the regulation of hematopoiesis via cytokines (PES = 4.55, *p *= 0.000346; Figure 8D, Table S1). The other two out of the five most enriched pathways for Calu-3 cells are free radical-induced apoptosis and the stress induction of HSP regulation (PES = 6.80, *p *= 3.21 × 10^−5^; Figure 8D, Table S2). These enriched pathways are associated with immune response and inflammation, which is also evidenced by our previous observations of enriched pathways identified based on their shared DEGs in AD patients, COVID-19 patients, and SARS-CoV-2-infected cells.

We also checked the most common enriched pathways shared between COVID-19 patients and SARS-CoV-2-infected cells, with a cut-off of PES > 2 and *p*-value < 0.05 (Figure 8E). The shared enriched pathways include hematopoietic cell lineage, the NOD-like receptor signaling pathway, Toll-like receptor signaling pathway, signal transduction through IL-1R, TNF signaling pathway, cytokine–cytokine receptor interaction, apoptosis, and osteoclast differentiation (Figure 8E). These commonly enriched pathways highlight the essential role of immune cell response and cytokines (such as IL-1), apoptosis, and the possible role of mitochondria (NOD-like receptor pathway) in both AD progression and SARS-CoV-2 infection.

## 4. Discussion

Recent research reported that COVID-19 may elevate the risk of cognitive impairment or even cause dementia in COVID-19 patients [18,19,21,88,89] and may possibly accelerate cognitive decline in AD patients [8,23]. Here, we investigated the potential molecular mechanisms underlying the interplay between COVID-19 and AD. Using comprehensive bioinformatics methods to analyze massive multi-omics datasets for COVID-19 patients, SARS-CoV-2-infected cell lines, and the gene expression data of AD patients, we identified the common differentially expressed genes (DEGs) after SARS-CoV-2 infection and in AD patients, and these DEGs are enriched in certain pathways such as immune responses and cytokine storms. We also constructed the gene interaction network with the signaling transduction module in the center, and *IRF7*, *STAT1*, *STAT2*, and *OAS1* were identified as the hub genes. We also checked the correlations between several major transcription factors and genes involved in the entry of SARS-CoV-2 into host cells. We observed that *ACE2* expression is positively correlated with *IRF7* expression in AD and coronavirus infections, and interestingly, *IRF7* is significantly up-regulated in response to different RNA virus infections. These findings could help us to examine the different possible mechanisms of the effect COVID-19 has on AD.

### 4.1. IRF7 and SARS-CoV-2 Entry into the Brain

The functions of the brain may be impaired if SARS-CoV-2 can directly invade the brain, and the most convincing model for the neuroinvasion of SARS-CoV-2 is through the olfactory route. In the hypothesis, the virus gains access to the CNS via the neural–mucosal interface in the olfactory mucosa and eventually reaches the olfactory bulb [7]. This supportive evidence includes the existence of SARS-CoV-2 RNA and the protein in human olfactory mucosa and the brain [26], human brain organoids, and mice brain [90]; independent autopsy studies also confirmed these observations [91], even in a 14-month-old child [92]. However, some pieces of evidence against the hypothesis are also present, and it is controversial whether SARS-CoV-2 can enter the brain directly or not. Some studies reported that SARS-CoV-2 was not detectable in the brain [34] or CSF [5]. Another evidence against the direct neuroinvasion hypothesis is that the expressions of both *ACE2* and *TMPRSS2* in the brains and neurons are very low, almost undetectable [7]. However, this evidence cannot rule out the possibility of entry through other key factors, such as *NRP1*, *BSG*, *CTSB*, and *FURIN*, and the expression levels of *BSG*, *NRP1*, and *FURIN* are relatively high in the prefrontal cortex regions [7]. Furthermore, even if the expression level of *ACE2* is relatively low in normal neurons, it could be up-regulated by other transcription factors in some conditions, such as in AD patients, which may help the neuroinvasion of SARS-CoV-2. In our research, we found that the expression of *ACE2* is up-regulated and positively correlated with the expression of *IRF7* in AD. Further analysis indicated that *IRF7* is significantly up-regulated upon different RNA virus infections, and *IRF7* may bind directly to the *ACE2* promoter to regulate the expression of *ACE2* in humans. *IRF7*, which belongs to the interferon regulatory factor family, regulates many IFN-α genes, and IFN-α has been proven to regulate the expression of *ACE2* [93]. Based on these observations, we proposed that *IRF7* can up-regulate the expression of *ACE2*, either directly (binding to the promoter) or indirectly (through IFN-α) in AD, which may help the brain’s entry of SARS-CoV-2. Our model raised another way to provide supportive evidence of a direct brain invasion.

### 4.2. IRF7 as a Major Player in Signaling Transduction

Interferon regulatory factors (IRFs) are transcription factors that regulate the transcription of interferons (IFNs). This family contains nine members (*IRF1-9*) in mammals, and *IRF7* is one of them [87]. The major function of *IRFs* is signaling transduction in innate immune responses [87,94,95,96]. During viral infection, IRF pathways, mainly *IRF3* and *IRF7*, are activated through signaling pathways triggered by different pattern-recognition receptors (PRRs), for example, transmembrane Toll-like receptors (TLRs), which can recognize the viral pathogen-associated molecular patterns (PAMPs) derived from invading pathogens [87,94,96]. *IRF7* has been reported to interact with *IRF3* and is the closest to *IRF3* in the IRF family [96,97]. *IRF3* has strict DNA-binding specificity (GAAANNGAAANN), while *IRF7* has broader DNA-binding specificity (GAAWNYGAAANY). We investigated the potential TFBSs in the promoter region of *ACE2* and predicted several *IRF3/IRF7*-binding sites in both human and mouse *ACE2*. *IRF3* is constitutively expressed in many cells, while the expression of *IRF7* is relatively low in most cell types and is only highly expressed in plasmacytoid dendritic cells (pDC), B cells, and monocytes [87]. Interestingly, at the later stages of viral infection, the expression of *IRF7* is significantly elevated via a positive feedback loop, in which type I *IFNs* induce *IRF7* and *IRF7* then induces expressions of several *IFNα* subtypes, such as *IFNα2*, *α5*, *α6*, and *α8* [98,99]. By binding to the IFN-α receptor (IFNAR), type I *IFNs* can activate the JAK-STAT signaling pathway, which is involved in immune response and cell death [94,99]. It has been reported that SARS-CoV-2 infection could elevate the expression levels of *IL-2/4/6/10*, *STAT1/2/3*, and *TNF-a* [7,37]. In this study, we constructed a gene interaction network with the signaling transduction module in the center, consisting of genes *IRF7*, *STAT1*, *STAT2*, and *OAS1*. We found that *IRF7* is significantly up-regulated in both AD and COVID-19 and, together with *STAT* genes (such as *STAT2*), are located in the core of the signaling transduction module. As a major player in the signaling transduction pathway, *IRF7* has been involved in the Toll-like receptor signaling pathway (the recognition of viral PAMPs), the *IFN* signaling pathway (*IRF7* positive feedback looping), and the innate immune system in both SARS-CoV-2 infection and AD.

### 4.3. Roles of IRF7 in Different Virus Infections and Other Diseases

*IRF7* was identified in 1997 in a latent Epstein–Barr virus (EBV) infection and was associated with the EBV latency program [100]. A homozygous deficiency of *IRF7* was reported to cause severe influenza and acute respiratory distress syndrome in a 2.5-year-old girl, which highlights the role of *IRF7* in severe influenza [101]. Few studies have focused on the roles of *IRF7* in coronavirus infections [102], and our analysis indicated that *IRF7* is significantly up-regulated upon different RNA virus infections, including SARS-CoV-2, SARS, MERS, and HIV. *IRF7* was recently found to be a mediator of protection and was correlated with the risk of HIV-1 acquisition [103]. We have also shown that *IRF7* expression is remarkably elevated in AD patients. A recent study surveyed several transcriptomic datasets from AD patients and observed that *IRF7* is up-regulated in some regions of the brain while it is also down-regulated in other regions in some samples [95], which implies that *IRF7* has cell-specific regulatory roles. Our analysis based on snRNA-seq data indicated that *IRF7* was up-regulated in NRGN neurons and macrophage cells upon SARS-CoV-2 infection. Besides the function of regulating immune responses through inducing type I *IFN* production, *IRF7* also plays critical roles in the regulation of oncogenesis, apoptosis, and cell differentiation [87,104,105]. As a common feature of massive neuronal death from apoptosis, apoptotic neurons and glial cells have been observed in AD [106,107]. Our analysis indicated that the common DEGs of COVID-19 and AD are enriched in the pathway of apoptosis, suggesting that apoptosis might be involved in COVID-19 and AD pathophysiology, and *IRF7* might play a critical role in apoptosis.

### 4.4. Epigenetic Regulation of IRF7 in SARS-CoV-2 Infection and AD Patients

As stated above, *IRF7* is relatively low in most types of cells, while it is highly expressed in plasmacytoid dendritic cells (pDC), B cells, and monocytes, which indicates that *IRF7* expression is cell-type specific. The cell-specific expression of *IRF7* may be regulated through epigenetic mechanisms, such as chromosomal accessibility [108] and DNA methylation [109]. The expression of *IRF7* could be silenced through the hypermethylation of the CpG islands in the promoter region of *IRF7* in human fibroblasts and 2fTGH [109]. In a recent epigenome-wide association study (EWAS), the authors detected the methylation statuses of CpG sites in *IRF7* via the methylation EPIC array in the peripheral blood samples of COVID-19 patients and found the different methylation statuses of CpG sites to be significantly associated with different disease severities [110]. In our study, the expression levels of *IRF7* are diverse in different cells and are upregulated upon different RNA virus infections, including SARS-CoV-2. It remains unclear whether the expression changes in *IRF7* in these cells are regulated by DNA methylation. In another independent study focusing on the methylomes of peripheral blood samples from COVID-19 patients after a 3-month recovery [111], we found that the DNA methylation status of *IRF7* has not been completely restored. These results indicate that epigenetic modifications may be involved in the regulation of *IRF7* in SARS-CoV-2 infection and AD and the detection of the methylation statuses of some factors in the blood, such as *IRF7*, which could provide insights for diagnosis and prognosis.

### 4.5. Immune Dysregulation and Neuroinflammation in AD Patients with COVID-19

A critical feature of the maladaptive immune response in COVID-19 is a hyperinflammatory response followed by immunosuppression [35,36,37]. In severe COVID-19 patients, the hyperinflammatory response, also called the cytokine storm, is a hallmark feature with elevated circulating levels of *IL-2/4/6/10*, *STAT1/2/3*, and *TNF-a* [7,37]. This systemic inflammation may further aggravate neuroinflammation and increase the susceptibility of patients to other neurological syndromes in AD. The systemic inflammatory mediators may access the CNS and cause damage through an impaired BBB function, which was reported in neurodegenerative diseases and viral infections, including SARS-CoV-2 infection [39,40]. Furthermore, microvascular injuries in the brain due to perfusion defects in SARS-CoV-2 infections were also reported [34]. Using intensive pathway analyses, we identified the enrichment of the *JAK-STAT* cascade, including cytokine signaling and *NF-kB* and *IFN* signaling pathways in both AD and COVID-19 patients. These findings may partly explain the cognitive decline in AD patients with COVID-19. As we reported, the expression levels of some key genes (including *IRF7*, *STAT2*, *OAS1*, *LCN2*, *LGAS9*, *DAPP1*, *IFITM2*, *TRIM21*, *CD55*, *S100P*, *IRAK3*, and *IFIH1*) were significantly up-expressed in both AD and COVID-19 patients, with some of them also being evidenced by several other studies [13,18,110], which highlights the critical roles of immune response factors in neuroinflammation in AD patients with COVID-19.

### 4.6. Mitochondrial Dysfunction in Neuropathogenesis of SARS-CoV-2 Infections

Mitochondrial dysfunction and the disturbance of energy production are proposed to trigger neurodegenerative diseases [112]. AD has been shown to be closely associated with various forms of mitochondrial dysfunctions, including low ATP production, excessive ROS production, calcium dyshomeostasis, and mitophagy [113]. Mitochondria-generated ROS and oxidized mtDNA could be used as endogenous DAMPs and trigger NLRP3 (nucleotide-binding oligomerization domain (NOD)-like receptor protein 3) inflammasome complex formation [80]. NLRP3 inflammasome activation could then contribute to the pathogenesis of AD by inducing and sustaining neuroinflammation [114]. Mitochondrial dysfunction induced by manipulations of host mitochondria using SRAS-CoV-2 viral open reading frames (ORFs) has also been reported [115]. A recent study reported that mitochondrial dysfunction, as the earliest feature of neurodegeneration, is a prelude to SARS-CoV-2-induced neuropathogenesis [79]. In our study, the common down-regulated DEGs shared in AD and COVID-19 patients are enriched in mitochondrion organization and the mitochondrial respiratory chain complex assembly. Furthermore, we found that the NOD-like receptor signaling pathway is a highly enriched pathway shared in AD patients, COVID-19 patients, and SARS-CoV-2-infected Calu-3 and NHBE cells. All of these findings are consistent with current research focusing on mitochondrial functions in AD and COVID-19, indicating that mitochondrial dysfunction may have very important functions in the neuropathogenesis of SARS-CoV-2.

All of these findings could improve our understanding of neurological manifestations in AD patients with SARS-CoV-2 infection. Considering the complex mechanisms underlying the interplay between COVID-19 and AD, including the potential neuro-invasion of SARS-CoV-2 and neuroinflammation triggered via different signaling pathways, it is necessary to investigate the mechanisms through laboratory and clinical collaborations. Based on our findings, physicians could develop guidelines for risk management and physical examinations in AD patients with SARS-CoV-2 infections, such as a pathogenic examination of cognitive decline and a test of serum levels of *IL-6* and *TNFa*. It is also essential to develop novel and effective therapeutics to prevent neurological damage and lower the risk of long-term cognitive decay.

## 5. Conclusions

Our comprehensive transcriptomic analysis provided not only possible supportive evidence of direct brain invasion but also robust evidence of neuroinflammation via the involvement of immune-related pathways after SARS-CoV-2 infection in AD patients. These findings could facilitate the investigation of the molecular mechanisms of the interplay between AD and COVID-19 and also develop effective therapeutic approaches for AD patients with COVID-19.

## Figures and Tables

**Table 1:** Datasets used in this study.

Disease Type	Dataset ID	Data Type	Tissues/Cells	Samples	Ref.
AD	GSE95587	RNA-Seq	Fusiform gyrus	117	Friedman BA et al. [43]
AD	GSE150696	Microarray	Brain	18	Low CYB et al. [44]
AD	GSE15222	Microarray	Cortical	363	Webster JA et al. [45]
AD	GSE1297	Microarray	Hippocampal	31	Blalock EM et al. [46]
AD	GSE33000	Microarray	Prefrontal cortex brain	467	Narayanan M et al. [47]
AD	GSE36980	Microarray	Hippocampi	18	Hokama M et al. [48]
AD	GSE37263	Microarray	Grey matter	16	Tan MG et al. [49]
AD	GSE29378	Microarray	Hippocampus	63	Miller JA et al. [50]
SARS-CoV-2	GSE147507	RNA-Seq	Calu-3	6	Blanco-Melo D et al. [42]
SARS-CoV-2	GSE147507	RNA-Seq	NHBE	6	Blanco-Melo D et al. [42]
SARS-CoV-2	GSE147507	RNA-Seq	Lung	4	Blanco-Melo D et al. [42]
SARS-CoV-2	GSE147507	RNA-Seq	A549	6	Blanco-Melo D et al. [42]
SARS-CoV-2	GSE152641	RNA-Seq	Whole Blood	86	Thair SA et al. [51]
SARS-CoV-2	GSE167000	RNA-Seq	Whole Blood	95	Galbraith MD et al. [52]
SARS-CoV-2	GSE182297	RNA-Seq	Brain	4	Pujadas E et al. [53]
SARS-CoV-2	GSE159812	snRNA-seq	Brain	30	Yang AC et al. [9]
SARS-CoV-2	GSE188847	RNA-Seq	Frontal cortex of brain	45	Mavrikaki M et al. [54]
SARS-CoV-2	GSE205099	RNA-Seq	Lung	16	Erjefält JS et al. [55]
SARS	GSE17400	Microarray	Calu-3	6	Yoshikawa T et al. [56]
SARS	GSE33267	Microarray	Calu-3	6	Sims AC et al. [57]
SARS	GSE45042	Microarray	Calu-3 2B4	6	Josset L et al. [58]
MERS	GSE122876	RNA-Seq	Calu-3	6	Yuan S et al. [59]
MERS	GSE65574	Microarray	Calu-3	6	Menachery VD et al. [60]
MERS	GSE81909	Microarray	Human airway epithelial cells	10	Feng S et al. [61]
HIV	GSE139327	RNA-Seq	PBMC	7	Muema DM et al. [62]
AD and SARS-CoV-2	GSE236562	RNA-Seq	Hippocampal formation	8	Griggs E et al. [63]

## Data Availability

No new data were created or analyzed in this study. Data sharing is not applicable to this article.

## Supplementary Materials

The following supporting information can be downloaded at: https://www.mdpi.com/article/10.3390/v16010100/s1, Figure S1: Enriched KEGG pathways for different groups of genes; Figure S2: *IRF7* plays a key role in pathways involved in signaling transduction in both AD and SARS-CoV-2 infection; Figure S3: The correlation between expressions of genes in core TFs andSARS-CoV-2 genes; Figure S4: *IRF7* is significantly up-regulated upon different RNA virus infections; Figure S5: The expression of *ACE2* is correlated with *IRF7*; Table S1: Details of the datasets used in this study; Table S2: PES for each Alzheimer’s disease-related pathway in each group.
