# Supplementary material for: Investigating the Potential Shared Molecular Mechanisms between COVID-19 and Alzheimer’s Disease via Transcriptomic Analysis
Source: Viruses. 2024 Jan 9;16(1):100. doi: 10.3390/v16010100 (PMC10821526; doi:10.3390/v16010100)
Supplement: Supplementary file 1 [file viruses-16-00100-s001.zip › Supplementary Figures.pdf]

viruses

MDPI

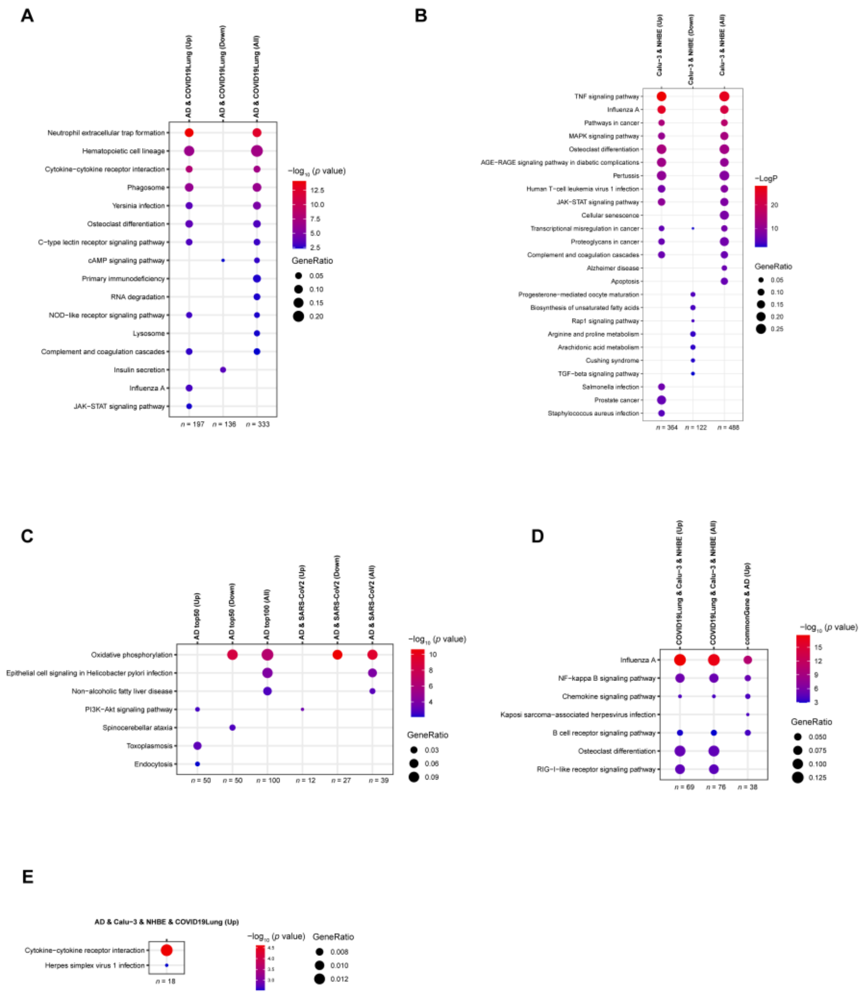

Figure S1. Enriched KEGG pathways for different groups of genes. (A) the same genes in Figure 2D; (B) the same genes in Figure 3D; (C) the same genes in Figure 4D; (D) the same genes in Figure 5D; (E) the same genes in Figure 6B.

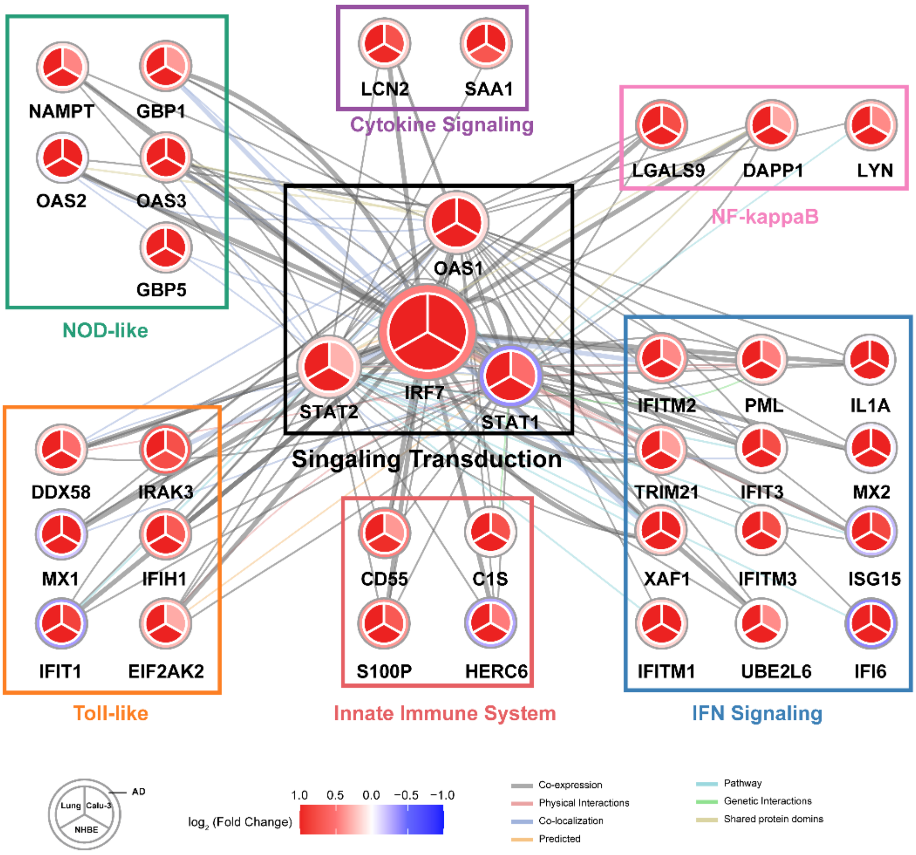

Figure S2. *IRF7* plays a key role in pathways involved in signaling transduction in both AD and SARS-CoV2 infection. The same node and network are identical to the ones in Figure 5C, with only the expression changes indicated by pie colors for SARS-CoV2 cells and COVID-19 patients, and the ring color for AD patients.

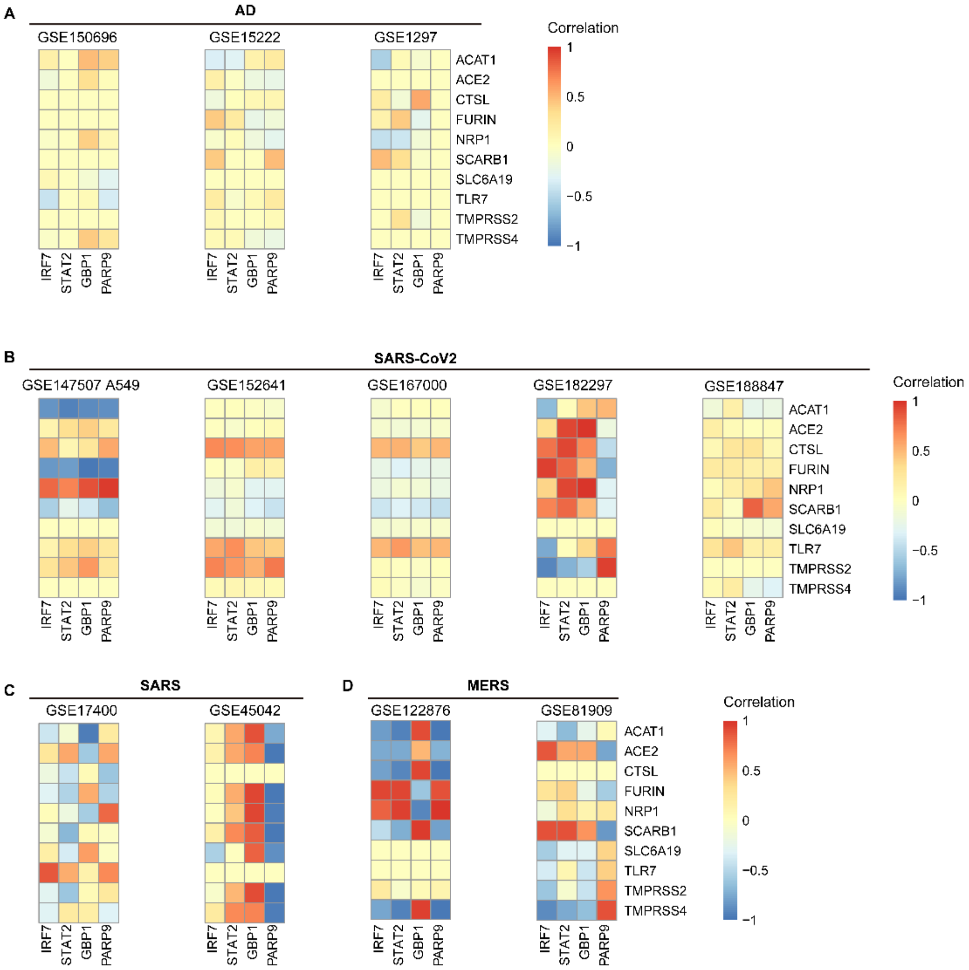

Figure S3. The correlation between expressions of genes in core TFs andSARS-CoV2 genes. Heatmaps show the gene expression changes in AD patients (A); SARS-CoV2 (B); SARS (C), and MERS (D) for the different groups of genes associated with SARS-CoV2 infection.

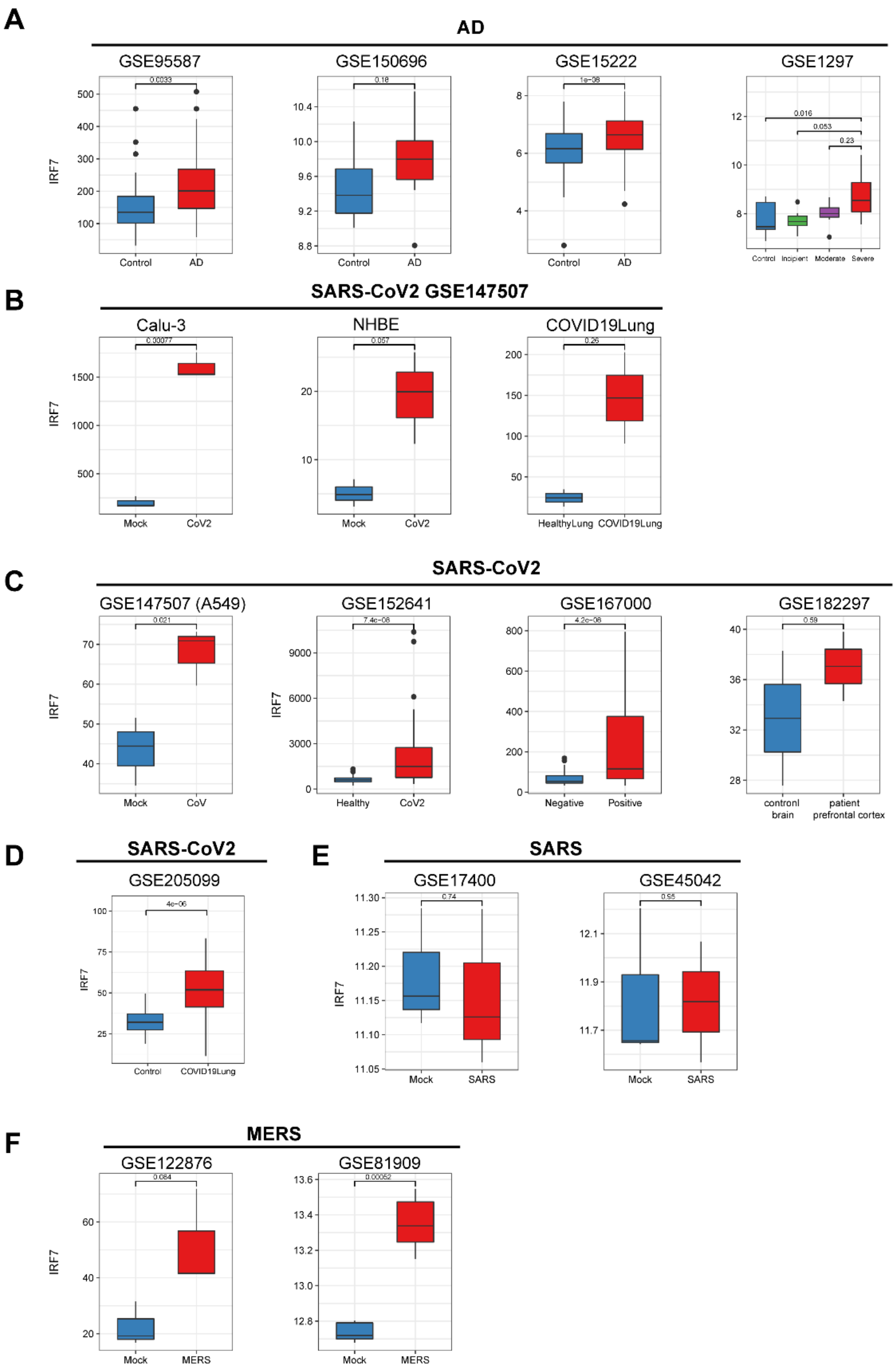

Figure S4. IRF7 is significantly up-regulated upon different RNA virus infections. The same box-plots as in Figure 7D, but for the other independent datasets: AD patients (A); SARS-CoV2 (B-D); SARS (E); MERS (F).

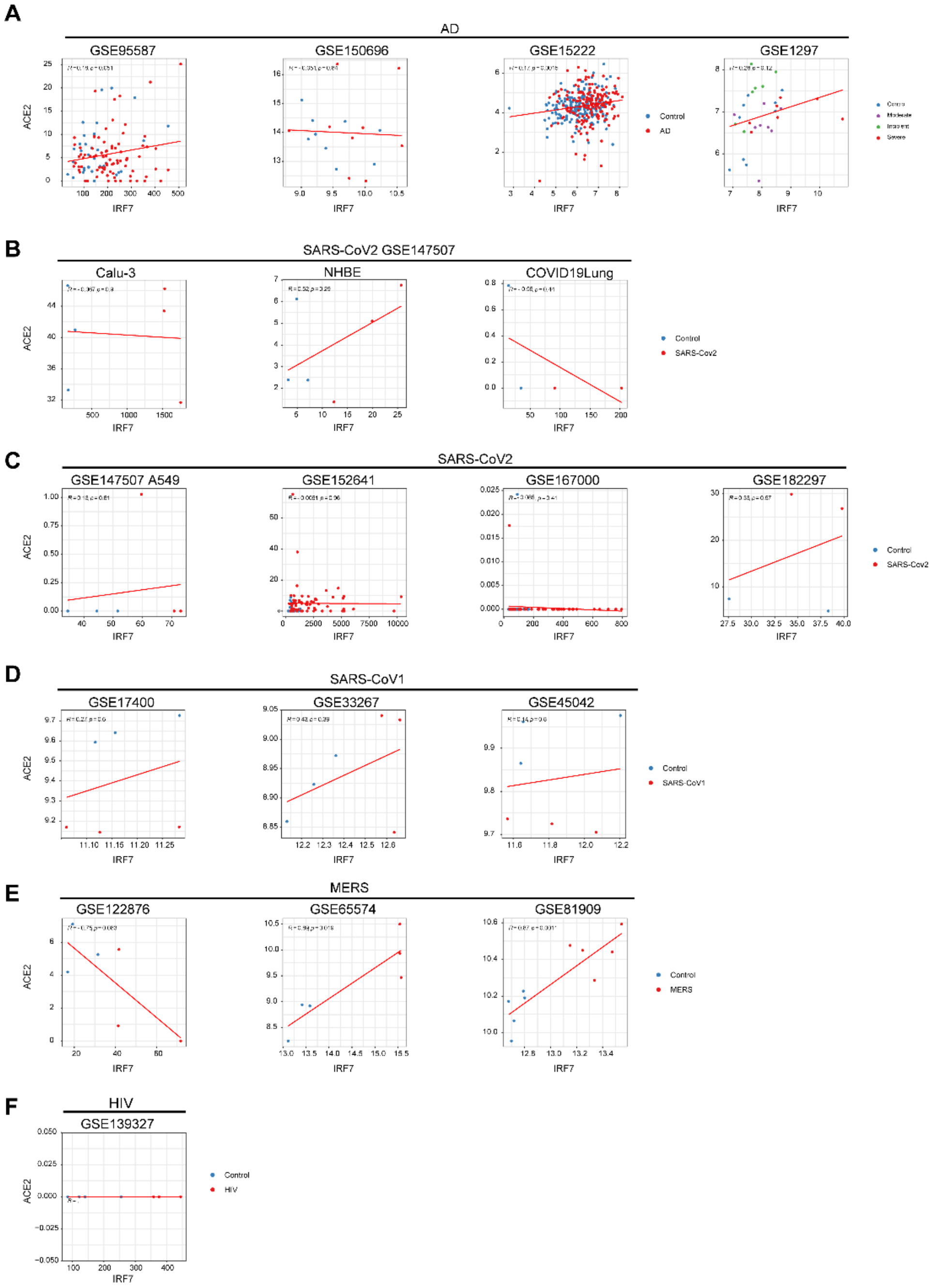

Figure S5. The expression of ACE2 is correlated with IRF7. The scatter plots of the expression of *ACE2* versus *IRF7* in AD patients (A), SARS-CoV2 (B-C), SARS-CoV1 (D), MERS (E), and HIV (F).
